# Magnitude and determinants of early initiation and exclusive breastfeeding at six weeks postpartum: evidence from the PMA Ethiopia longitudinal survey

**DOI:** 10.1186/s13006-023-00611-y

**Published:** 2024-01-04

**Authors:** Addisalem Zebene Armdie, Bedilu Alamirie Ejigu, Assefa Seme, Selamawit Desta, Mahari Yihdego, Solomon Shiferaw

**Affiliations:** 1 PMA Ethiopia, Addis Ababa University, Addis Ababa, Ethiopia; 2 Department of Statistics, College of Natural and Computational Sciences, Addis Ababa University, Addis Ababa, Ethiopia; 3 School of Public Health, Addis Ababa University, Addis Ababa, Ethiopia; 4 Department of Population Family and Reproductive Health, Johns Hopkins Bloomberg School of Public Health, Baltimore, MD USA

**Keywords:** Exclusive breastfeeding, Timely initiation breastfeeding, Ethiopia, Prevalence, Factors, Longitudinal data

## Abstract

**Background:**

Early initiation and exclusive breastfeeding are crucial in preventing child morbidity and mortality. Despite the importance of these practices, rates of timely initiation of breastfeeding and exclusive breastfeeding remain suboptimal in many sub-Saharan countries, including Ethiopia. This paper aimed to estimate the prevalence and identify determinants of breastfeeding initiation within the first hour after birth and exclusive breastfeeding in Ethiopia.

**Methods:**

Data from the Performance Monitoring for Action Ethiopia project, a national survey conducted from August 2019 to September 2020, were used. The analytical sample comprises 2564 postpartum women and their children; data reported at baseline during enrollment and six weeks postpartum were used in this analysis. A multi-level binary logistic regression model was employed to identify determinant factors linked with initiation breastfeeding and exclusive breastfeeding.

**Results:**

Of the 2564 mothers, 77.8% of infants breastfed within the first hour of birth and 68.4% of women practiced exclusive breastfeeding at six weeks postpartum with significant variation across regions. In the multivariate analysis, mothers who had cesarean delivery were less likely to initiate early breastfeeding as compared to mothers with vaginal delivery (AOR 0.27; 95% CI 0.17, 0.41). The odds of early initiation of breastfeeding were higher for mothers whose baby cried immediately after birth (AOR 3.31; 95% CI 1.95, 5.62) and who had skilled assisted delivery (AOR 2.13; 95% CI 1.01, 4.48). Other factors that were significantly associated with early initiation of breastfeeding were obstetric complication(s), parity, immediate mother-to-baby skin-to-skin contact, immediate postnatal care and the region. Similarly, mothers whose babies had a good neonatal birth status (AOR 1.81; 95% CI 1.09, 2.99) were more likely to exclusively breastfeed their child at six weeks postpartum.

**Conclusions:**

Early initiation of breastfeeding and exclusive breastfeeding is sub-optimal in Ethiopia. Nutrition programmers should consider regional variations in designing intervention programs to enhance breastfeeding practices. Healthcare providers should give special attention to women at risk such as those giving birth through cesarean section and having obstetric complications during delivery.

## Background

Since 1990, there has been a significant improvement in child survival worldwide. However, the decrease in neonatal death from 5 million in 1990 to 2.4 million in 2020 has been slower than the decrease in post-neonatal under-5 mortality [1]. Addressing mortality during the neonatal period requires a system-wide approach and a set of interventions including promoting breastfeeding within the first hour after birth and exclusive breastfeeding for the first six months postpartum [2, 3].

The World Health Organization (WHO) recommends that all neonates be breastfed within one hour of birth which is defined as early initiation of breastfeeding (early initiation of breastfeeding) and be exclusively breastfed (EBF) as the infant receiving only breast milk, no other liquids or solids with the exception of oral rehydration solution, supplements or medicines up to six months of age [4].

Early initiation of breastfeeding provides the infant with colostrum. Since the first milk is rich in anti-microbial and anti-inflammatory agents such as lactobacillus bifidus, lactoferrin, and secretory immunoglobulin A (sIgA) which can protect babies from infections, it is often called the “first immunization” [5]. Early breastmilk intake also encourages the maturity of the intestines, and introduces new microbial communities that stimulate growth and development of the infant gut microbiome [6]. In addition, at the time of initiation of early breastfeeding, there is skin-to-skin contact between the baby and mother, and the skin temperature of the mother's chest will adjust to the baby's body temperature, thereby reducing the risk of hypothermia [7].

Exclusive breastfeeding or feeding only breast-milk eliminates the ingestion of pathogenic micro-organisms through contaminated water, other fluids, and foods, and also prevents damage to the immunologic barriers in the infant’s gut from contaminants or allergenic substances in infant formula or food [8]. Babies who receive early initiation of breastfeeding, and who are breastfed exclusively for the first six months are less likely to experience gastrointestinal and respiratory infection.

According to an analysis of the Demographic and Health Survey (DHS) in Sub-Saharan Africa and South Asian countries, the prevalence of early initiation of breastfeeding among children ranged from 34.7% to 66.4%, with the lowest percentage in Nigeria and the highest in Nepal [9–12]. As per a meta-analysis done using 29 sub-Saharan African countries the overall prevalence of exclusive breastfeeding ranged between a lowest of 23.70% in Central Africa to a highest of 56.57% in Southern Africa [13]. In Ethiopia, according to 2016 Ethiopian Demographic and Health Survey (EDHS), less than three-quarter (73%) of infants had timely initiation of breastfeeding and 58% of children under six months of age were exclusively breastfed [14].

Various studies from different countries have identified different factors affecting timely initiation and exclusive breastfeeding [15, 16]. A recent systematic review in South Asia, which included 25 studies from 7 countries, revealed that early initiation of breastfeeding is predominately associated with socioeconomic, health-related and individual factors; newborn sex, maternal age, presence of a professional birth attendant, maternal education, parity, caesarean section and low birthweight [17]. A pooled data from the DHS program in 27 Sub-Saharan countries showed that mothers with a secondary education or higher, mothers within the ages of 25–34 years, rural residence, richer household wealth quantile, 4 + antenatal care visit, delivering in a health facility, singleton birth, being a female infant and younger age infants were the factors that were associated with increased likelihood of exclusive breastfeeding [18].

Most of the studies on this topic in Ethiopia and other African countries use data from relatively small-scale surveys. Studies using large scale survey like the EDHS ask women who have children 0–23 months of age and therefore rely on mother’s recall of timing of initiation and exclusive breastfeeding practice and as such, they may not provide accurate data on either the population-based prevalence, or barriers to early initiation of breastfeeding and exclusive breastfeeding.

To address these methodological limitations and gaps in the current global evidence-base, we analyzed the Performance Monitoring for Action (PMA) Ethiopia cohort survey data which prospectively collect data from all women who are pregnant or six weeks postpartum at the time of enrollment in the survey and follows up with these women at 6-weeks, 6-months, and 1-year postpartum, nationally. The data at six-week postpartum enabled us to determine the rate and contributing factors of early initiation of breastfeeding and EBF, when mothers may more easily recall their breastfeeding behavior. A further benefit of the study's panel design enabled us to investigate additional maternal characteristics that are not covered by previous cross-sectional studies, such as maternity waiting home use, newborn outcome (need of resuscitation or not) and mother to baby skin-to-skin contact.

Therefore, this study aims to determine the prevalence and determinants of early initiation of breastfeeding and exclusive breastfeeding at six weeks postpartum, using national panel survey data conducted in Ethiopia.

## Methods

### Data source

This analysis used data from PMA Ethiopia, a cohort study conducted from October 2019 to August 2021. PMA Ethiopia survey is a national study being implemented in collaboration between Addis Ababa University (AAU), the Ethiopia Federal Ministry of Health (FMOH), and Johns Hopkins Bloomberg School of Public Health (JHSPH). PMA Ethiopia generates timely cross-sectional and longitudinal data on reproductive, maternal, and newborn health indicators to inform national and regional government priorities and policies [19].

PMA Ethiopia conducted a full census in 217 enumeration areas (EA) across six regions and listed 36,614 households between October and November 2019. All women aged 15–49 in these households were screened (32,792) and, if they reported being currently pregnant or having delivered within the past six weeks, were eligible for the panel study; 2,889 women were identified as eligible and 2,855 enrolled with a response rate of 98.4% and 93.6% for the baseline and 6-week surveys, respectively. The panel was conducted in six regions that collectively represent 90% of the population in Ethiopia; Addis Ababa, Afar, Amhara, Oromia, SNNP and Tigray [19].

The baseline interview collected information about women’s sociodemographic characteristics. Information about initiation of breastfeeding, exclusive breastfeeding and maternal health service-related variables was collected at the six-week postpartum follow-up interview. Women who were already 5–9 weeks postpartum at enrollment were asked all the sociodemographic, breastfeeding and maternal health service-related questions during the baseline interview. Informed consent to participate was obtained at the screening and prior to enrollment by all participants and was provided verbally [19].

**Fig. 1:**
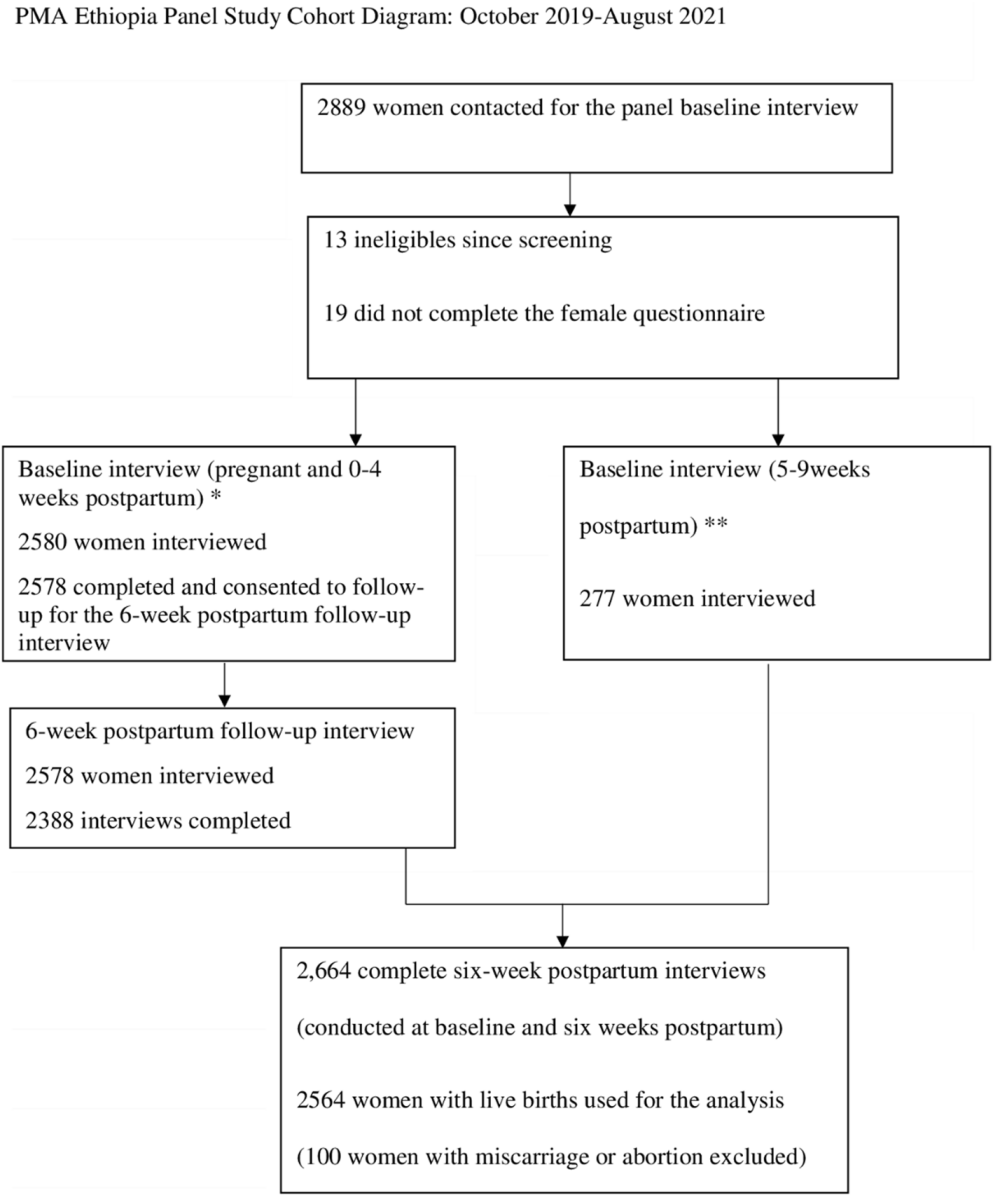
PMA Ethiopia Panel study cohort diagram; October 2019 to August 2021

Among the 2855 enrolled pregnant or recently postpartum women in our survey, 2,664 completed six-week postpartum interviews (conducted at baseline and six weeks postpartum), 2564 women with live births used for the analysis (100 women with miscarriage or abortion excluded) (Fig. 1).

Estimated or actual delivery dates of women were used to schedule the six-week postpartum interview, which was conducted when respondents were about six weeks postpartum. *Women who were pregnant or 0–4 weeks postpartum at the time of the first panel interview received the sociodemographic characteristics related survey questions at baseline and questions related to their breastfeeding behavior and maternal care services at 6-week postpartum interview. **Women who were 5–9 weeks postpartum at the time of the first panel interview received a combined set of survey questions that included the six-week survey, that other women received during two separate interviews.

We used the baseline and six-week postpartum interview data that are collected at that particular period in order to assess the magnitude of early initiation of breastfeeding and exclusive breastfeeding and its relationship with the sociodemographic, maternal and child health related factors.

### Study variables

Early initiation of breastfeeding and exclusive breastfeeding (EBF) are the main outcomes (variables of interest) of this study.

Early initiation of breastfeeding was assessed by asking women *“How long after birth did you first put the baby to the breast”*, responses were recorded in minutes, hours, or days when baby was first put to breast. A binary variable was then created and categorized as: (1) early initiation of breastfeeding if a newborn was breastfed within 1 h after birth, or (2) delayed initiation of breastfeeding if a child was breastfed after 1 h [20]. This indicator was self-reported by mothers who were approximately six-weeks postpartum at time of interview.

Exclusive breastfeeding was calculated as the proportion of infants, who received only breast milk and no other liquids or solids based on a 24-h recall (exclusive of medication, immunizations, ORS drops, or vitamin supplements) at the 6-week follow-up visit. Similarly, exclusive breastfeeding was expressed as a dichotomous variable with category 1 for EBF and category 0 for non-EBF [19].

Table 1 shows the operational definition/ description for all the variables included in this study.

**Table 1:** Operational definition of variables

	**Variable description**
Maternal age	Classified as: 15–19, 20–24, 25–34, 35–49
Parity	Number of births: 0 births, 1–2 births, 3 + births
Education	Never attended, primary, secondary/higher
Marital status	Not married, Married/in union, Single/divorced/widowed
Religion	Protestant, Orthodox, Muslim, catholic/others
Place of delivery	Facility, Home
Mode of delivery	Caesarean, Vaginal
Type of delivery attendant	Skill birth attendant (Yes/No)
Maternity home use	Mother use maternity waiting home before labor (Yes/No)
Child sex	Male, female
Household wealth index	Household Wealth quintiles (low, middle, high)
Residence	Place of residence: urban, rural
Region	Six administrative regions of Ethiopia where the PMA Ethiopia panel survey is conducted (Addis Ababa, Afar, Amhara, Oromia, SNNP, and Tigray)
PNC	Mothers are considered to have PNC, if they have immediate visit with in 1 h of birth (for the early initiation of breastfeeding) or PNC visit at six-week postpartum period (for EBF)
Pregnancy intention	Mothers who want to become pregnant then (intended) or want to wait until later or not want to have any / any more children at all (unintended) at the time she became pregnant
Postpartum nutrition counseling	If Mothers got a counseling about EBF during PNC visit from health care providers (Yes/No)
Obstetric Complication	Presence of any of the below self-reported maternal complications. This are “Bleeding, membrane rupture before labor, membrane rupture at < 9 months gestation, malpresentation/malposition, prolonged labor (> 12 h), convulsion, retained placenta (> 30 min),high fever with foul smelling discharge or lower abdominal pain”
Immediate neonatal birth status	Whether baby cried immediately after birth or baby needed Resuscitation after birth
Immediate skin-to-skin contact	Whether baby placed to mother’s chest immediately after birth or not
Child illness	Whether baby suffered from any illness since birth

### Statistical analysis

We used descriptive statistics to examine the distribution of women’s sociodemographic and reproductive characteristics (Table 2, pattern of initiation of breastfeeding and exclusive breastfeeding). We assessed the bivariate distributions of early initiation of breastfeeding and exclusive breastfeeding with each covariate. Maternal and infant characteristics of early initiation of breastfeeding and EBF were compared to those of breastfeeding initiation > 1 h after birth, and non EBF respectively.

**Table 2:** Individual, household and community level characteristics of living infants aged 6 weeks, PMA Ethiopia 2019–2021 cohort (*n* = 2564)

**Variables**	**Overall**	
	***n***	**%**
**Maternal age (in years)**		
15–19	273	10.7
20–24	621	24.2
25–34	1252	48.8
**35–49**	418	16.3
**Maternal education**		
Never attended	1052	41.0
Primary	1024	39.9
Secondary /higher	487	19.0
**Marital status**		
Married	2443	95.3
Unmarried/divorced/widowed	121	4.7
**Residence**		
Urban	584	22.8
Rural	1980	77.2
**Region**		
Addis	103	4.0
Afar	50	2.0
Amhara	527	20.6
Oromia	1121	43.7
SNNP	584	22.8
Tigray	178	7.0
**Religion**		
Protestant	647	25.2
Orthodox	1001	39.0
Muslim	867	33.8
Catholic/other	49	1.9
**Parity**		
1	1028	40.1
2 +	1536	59.9
**Wealth quintile**		
Low	1029	40.1
Middle	508	19.8
High	1027	40.1
**Antenatal care visits**		
Zero visit	798	31.1
1–3 visits	796	31.1
4 or more visits	970	37.8
**Delivery place**		
Home	1169	45.6
Facility	1395	54.4
**Mode of delivery**		
Vaginal	2423	94.5
Caesarean	141	5.5
**Skilled birth attendant**		
**Yes**	1502	58.6
**No**	1062	41.4
**Maternity waiting home use before labor**		
Yes	378	14.7
No	2186	85.3
**PNC**		
No	1688	65.8
Yes	876	34.2
**Discussion about EBF during PNC**		
Yes	371	14.5
No	2193	85.5
**Obstetric complication**		
Yes	956	37.3
No	1608	62.7
**Pregnancy intention**		
Intended	1629	63.5
Unintended	935	36.5
**Newborn sex**		
Male	1310	52.0
Female	1210	48.0
**Birth outcome**		
Single	2521	98.3
Twin	43	1.7
**Baby cry/breathe normally immediately after birth**		
**Yes**	2446	95.4
**No**	118	4.6
**Baby placed on mother’s chest immediately after delivery**		
Yes	1150	44.9
No	1414	55.1
**Infant illness since birth**		
No illness	1517	59.2
Any illness	1047	40.8

We conducted a series of bivariate and multivariate analyses by using a multilevel binary logistic regression model to examine the association between explanatory variables with the outcome variables while controlling for confounding factors. Those explanatory variables that showed significance (*p* < 0.25) in the bivariate analysis were included for multivariate analysis. Let ${y}_{ij}$ denote the binary outcome for subject *i* in cluster j, and assume ${y}_{ij}$ follows a Bernoulli distribution with probability of success, ${p}_{ij}$. Then, using the usual logit link function, a binary outcome can be associated with a linear predictor as follows:

$$logit\left({p}_{ij}\right)={\beta }_{0}+\beta {X}_{ij}+{u}_{j}$$

where ${\beta }_{0}$ is an intercept, $\beta$ is an unknown parameter for individual level predictors, and ${u}_{j}$ are mutually independent Gaussian random effects used to capture within-cluster correlation. In standard multilevel models, ${u}_{j}$ is usually assumed to be a normally distributed random intercept with mean 0 and variance ${\sigma }_{u}^{2}$. The multilevel approach produces reliable standard errors and parameter estimates when outcomes for individuals within clusters are correlated [21, 22].

Breastfeeding initiation at > 1 h after birth, and non-exclusive breastfeeding respectively and multiple logistic regression analysis was performed to determine the adjusted odds ratio (AOR). We used multivariate, multilevel models to estimate differences in women’s odds of timely initiation of breastfeeding and exclusive breastfeeding by individual characteristics, adjusting for clustering of women within the enumeration area. We examined model fit by analyzing model fit statistics (i.e., Aikake’s Information Criterion (AIC) values) and assessed collinearity at 0.6 using a correlation matrix for all analytic variables. Statistical significance for the adjusted multivariate analysis was set to *p* < 0.05. All analyses were weighted to reflect the national population of pregnant and postpartum women in Ethiopia and to account for the complex survey design. All analyses were conducted in Stata/SE, Version 17 [23].

### Ethical considerations

The PMA Ethiopia survey received ethical approval from Addis Ababa University, College of Health Sciences (AAU/CHS) (Ref: AAUMF 03–008) and the Johns Hopkins University Bloomberg School of Public Health (JHSPH) Institutional Review Board (FWA00000287). Additional information on the PMA Ethiopia survey design, including weighting and informed consent procedures, is described elsewhere [19].

## Results

### Sociodemographic and maternal health service characteristics

Tables 2 and 3 presents the sociodemographic characteristics of the mothers/infants and the distribution of breastfeeding practices by background characteristics. A total of 2564 mothers who are at six weeks postpartum were included in the study, and 48.8% of the women were between 25–34 years old. Four in every ten mothers (41.0%) had never attended school. A predominant percentage of mothers lived in rural area (77.2%) and in Oromia region (43.7%).

**Table 3:** Percent distribution of early initiation of breastfeeding and EBF by background characteristics, among infants six weeks old and still alive at the time of the interview, PMA Ethiopia 2019–2021 cohort (*n* = 2564)

Background characteristics	Time to First Breastfeed		*P* value	Exclusively breastfed		*P* value
	**Early** **n (%)**	**Delayed** **n (%)**		**Yes** **n (%)**	**No** **n (%)**	
**Maternal age (in years)**						
15–19	207 (75.8%)	66 (24.2%)	0.067	165 (60.4%)	108 (39.6%)	0.022*
20–24	504(81.3%)	116 (18.7%)		440 (70.9%)	181 (29.1%)	
25–34	972(77.7%)	280 (22.3%)		864 (69.0%)	388 (31.0%)	
**35–49**	312(74.6%)	106 (25.4%)		285 (68.2%)	133 (31,8%)	
**Maternal education**						
Never attended	813 (77.2%)	240 (22.8%)	0.001*	751 (71.4%)	302 (28.6%)	0.101
Primary	781 (76.3%)	243 (23.7%)		660 (64.4%)	364 (35.6%)	
Secondary /higher	402 (82.5%)	85 (17.5%)		343 (70.4%)	144 (29.6%)	
**Marital status**						
Married	1907 (78.0%)	536 (22.0%)	0.795	1666 (68.2%)	777 (31.8%)	0.465
Unmarried/divorced/widowed	89 (73.7%)	32 (26.3%)		88 (72.6%)	33 (27.4%)	
**Residence**						
Urban	474 (81.2%)	110 (18.8%)	0.000*	394 (67.5%)	190 (32.5%)	0.244
Rural	1521 (76.8%)	459 (23.2%)		1360(68.7%)	621 (31.3%)	
**Region**						
Addis	82 (79.0%)	22 (21.0%)	0.000*	59 (56.7%)	45 (43.3%)	0.000*
Afar	32 (64.3%)	18 (35.7%)		24 (47.5%)	26 (52.5%)	
Amhara	377 (71.4%)	151 (28.6%)		405 (76.8%)	122 (23.2%)	
Oromia	921 (82.1%)	200 (17.9%)		776 (69.2%)	345 (30.8%)	
SNNP	456 (78.1%)	128 (21.9%)		347 (59.4%)	237 (40.6%)	
Tigray	128 (72.0%)	50 (28.0%)		144 (80.7%)	34 (19.3%)	
**Religion**						
Protestant	507 (78.4%)	140 (21.6%)	0.578	414 (64.0%)	233 (36.0%)	0.000*
Orthodox	735 (73.4%)	266 (26.6%)		724 (72.4%)	277 (27.6%)	
Muslim	713 (82.3%)	153 (17.7%)		583 (67.3%)	283 (32.7%)	
Catholic/other	40 (81.6%)	9 (18.4%)		32 (64.6%)	18 (35.4%)	
**Parity**						
			0.467			0.019
1	781 (76.02%)	247 (23.98%)		676 (65.7%)	352(34.3%)	
2 +	1996 (77.84%)	568 (22.16%)		1078 (70.2%)	458 (29.8%)	
**Wealth quintile**						
Low	789 (76.7%)	239 (23.3%)	0.002*	694 (67.4%)	335 (32.6%)	0.066
Middle	396 (78.0%)	112 (22.0%)		354 (69.7%)	154 (30.3%)	
High	810 (78.9%)	217 (21.1%)		706 (68.7%)	321 (31.3%)	
**Antenatal care visits**						
Zero visit	614 (77.0%)	184 (23.0%)	0.009*	506 (63.4%)	292 (36.6%)	0.000*
1–3 visits	613 (76.9%)	184 (23.1%)		572 (71.8%)	224 (28.2%)	
4 or more visits	769 (79.3%)	201 (20.7%)		676 (69.7%)	294 (30.3%)	
**Delivery place**						
Home	864 (73.9%)	306 (26.1%)	0.000*	801 (68.5%)	368 (31.5%)	0.139
Facility	1132 (81.2%)	263 (18.8%)		953 (68.3%)	442 (31.7%)	
**Mode of delivery**						
Vaginal	1915 (79.0%)	509 (21.0%)	0.000*	1666 (68.7%)	757 (31.3%)	0.112
Caesarean	81 (57.7%)	60 (42.3%)		88 (62.5%)	53 (37.5%)	
**Skilled birth attendant**						
Yes	1219 (81.2%)	283 (18.8%)	0.000*	1026 (68.3%)	476 (31.7%)	0.178
No	776 (73.1%)	285 (26.9%)		727 (68.5%)	334 (31.5%)	
**Maternity waiting home use before labor**						
Yes	296 (78.5%)	81 (21.5%)	0.062	263 (69.8%)	114 (30.2%)	0.019*
No	1699 (77.7%)	487 (22.3%)		1490 (68.2%)	696 (31.8%)	
**PNC**						
No	1284 (76.0%)	405 (24.0%)	0.000*	1167 (69.1%)	587 (67.0%)	0.492
Yes	712 (81.3%)	163 (18.7%)		587 (67.0%)	289 (33.0%)	
**Discussion about EBF during PNC**						
Yes	287 (77.3%)	84 (22.7%)	0.388	250 (67.2%)	122 (32.8%)	0.784
No	1709 (77.9%)	484 (22.1)		1504 (68.6%)	688 (31.4%)	
**Obstetric complication**						
Yes	694 (72.6%)	262 (27.4%)	0.000*	635 (66.4%)	322 (33.6%0	0.089
No	1302 (81.0%)	306 (19.0%)		1119 (69.6%)	489 (30.4%)	
**Pregnancy intention**						
Intended	1279 (78.5%)	350 (21.5%)	0.979	1114 (68.4%)	515 (31.6%)	0.900
Unintended	717 (76.7%)	218 (23.3%)		640 (68.4%)	295 (31.6%)	
**Newborn’s sex**						
Male	1028 (78.5%)	282 (21.5%)	0.314	875 (66.8%)	435 (33.2%)	0.000*
Female	967 (79.9%)	244 (20.1%)		878 (72.6%)	332 (27.4%)	
**Birth outcome**						
Single	1968 (78.1%)	552 (21.9%)	0.002*	1751 (69.5%)	770 (30.5%)	0.000*
Twin	28 (63.4%)	16 (36.6%)		3 (7.0%)	40 (93.0%)	
**Baby cry/breathe normally immediately after birth**						
Yes	1952 (79.8%)	494 (20.2%)	0.000*	1707 (69.8%)	740 (30.2%)	0.000*
No	43 (36.9%)	74 (63.1%)		47 (40.2%)	70 (59.8%)	
**Baby placed on mother’s chest immediately after delivery**						
Yes	976 (84.8%)	174 (15.2%)	0.000*	813 (70.7%)	337 (29.3%)	0.000*
No	1020 (72.1%)	394 (27.9%)		941 (66.6%)	473 (33.4%)	
**Infant illness since birth**						
No illness	1193 (78.6%)	325 (21.4%)	0.039*	1104 (72.7%)	414 (27.3%)	0.000*
Any illness	803 (76.7%)	244 (23.3%)		650 (62.1%)	397 (37.9%)	

^*^P value < 0.05

**Fig. 2:**
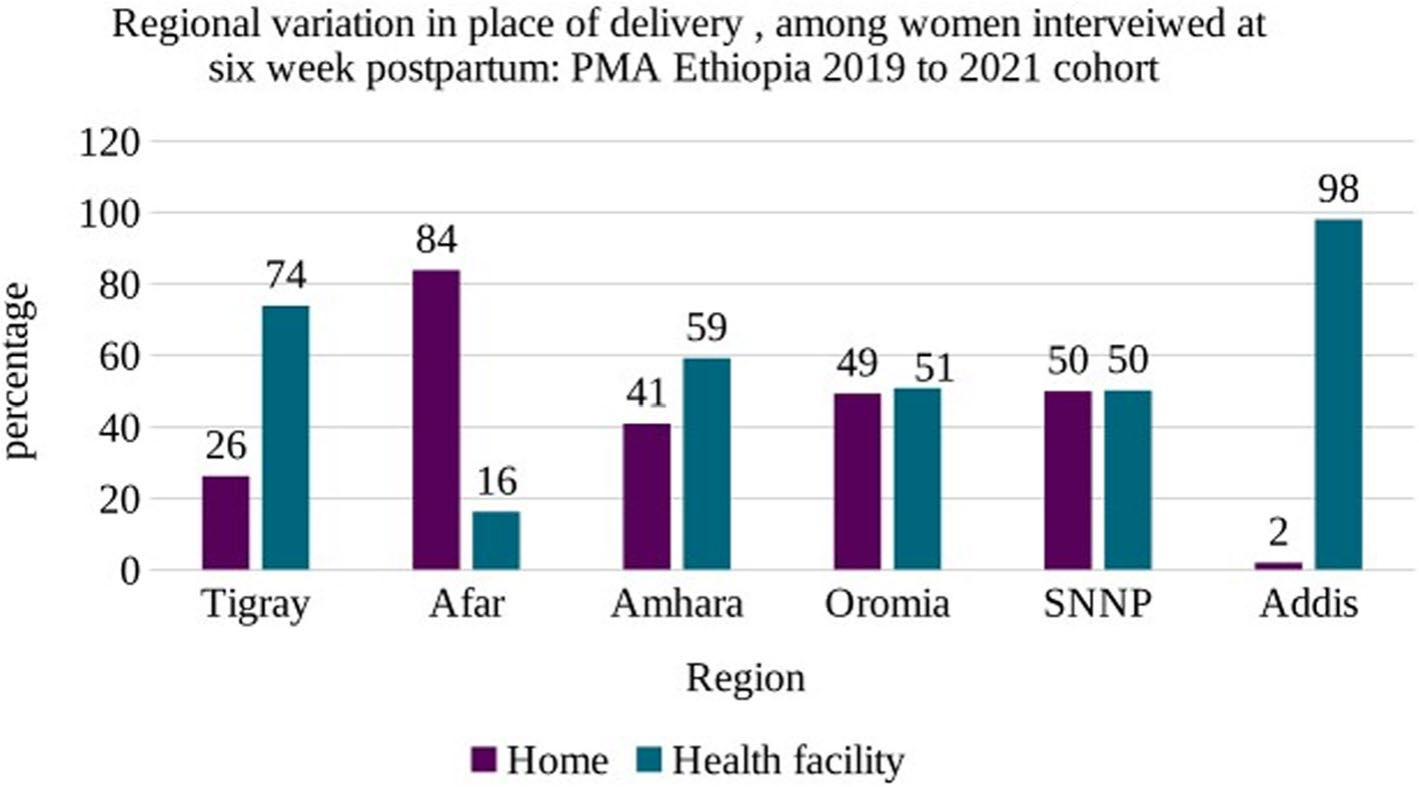
Regional variation in place of delivery, among women interviewed at six weeks postpartum: PMA Ethiopia 2019 to 2021 cohort

More than half (54.4%) of women delivered at a health facility with regional variation (Fig. 2) The majority (94.5%) of the women had vaginal delivery for their most recent birth and approximately six in ten (58.6%) women’s deliveries were assisted by a skilled birth attendant, and 37.3% of the respondents reported having had some complications during or within 24 h of delivery.

Most of the women (95.4%) reported that their baby cried/breathed normally immediately after birth without need of any resuscitation. Less than half of newborns (44.9%) were placed on their mother’s chest immediately after birth. More than three fifth (65.8%) of the mothers reported not having any postnatal care (PNC) after delivery and only 14.5% of women reported that the health provider discusses about exclusive breastfeeding during their PNC visit. Four in ten (40.8%) infants had experienced some illness since delivery.

### Breastfeeding characteristics

**Fig. 3:**
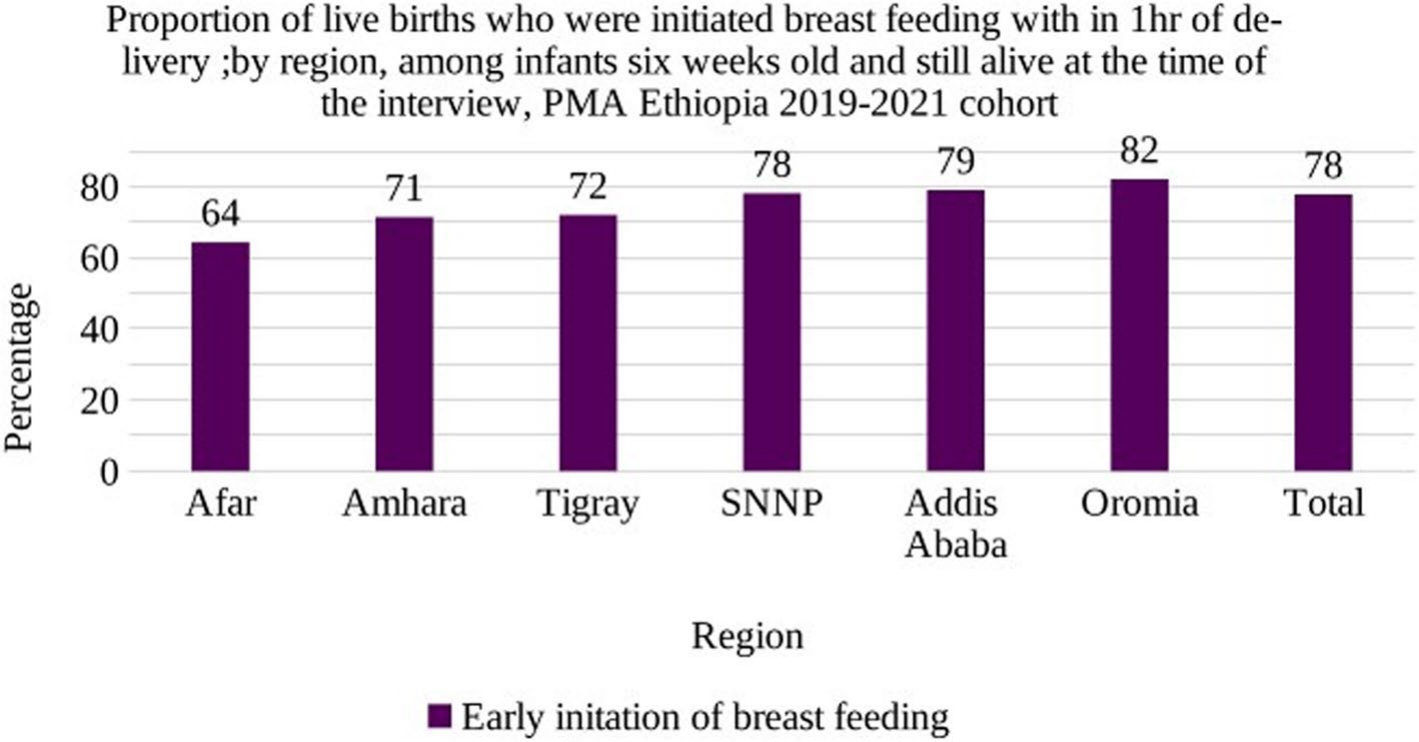
Proportion of live births who were initiated breastfeeding with in 1 h of delivery, by region: among infants six weeks old and still alive at the time of the interview, PMA Ethiopia 2019–2021 cohort

The majority of the respondents (93.3%) reported that they were breastfeeding during the six-week postpartum interview. Of the total 2564 enrolled six-week postpartum women, 77.8% of mothers reported breastfeeding their infants within first hour of birth. The percentage of timely initiation of breastfeeding is higher in Oromia followed by Addis Ababa and lowest in Afar region (Fig. 3).

About a quarter of the surveyed women-initiated breastfeeding between one and 24 h of birth and 7% started breastfeeding after 24 h of delivery (Table 4).

**Table 4:** Children’s breastfeeding initiation time, among infants six weeks old and still alive at the time of interview, PMA Ethiopia 2019–2021 cohort (*n* = 2564)

Time to breastfeeding initiation	n	%
Within 1 h of delivery	1996	77.8
1 h to 24 h. of delivery	648	25.3
24 h. to 48 h. of delivery	103	4.00
> 48 h of delivery	74	2.9
BF not initiated by the time of 6 week survey	180	7.0
**Total**	**2564**	**100**

**Fig. 4:**
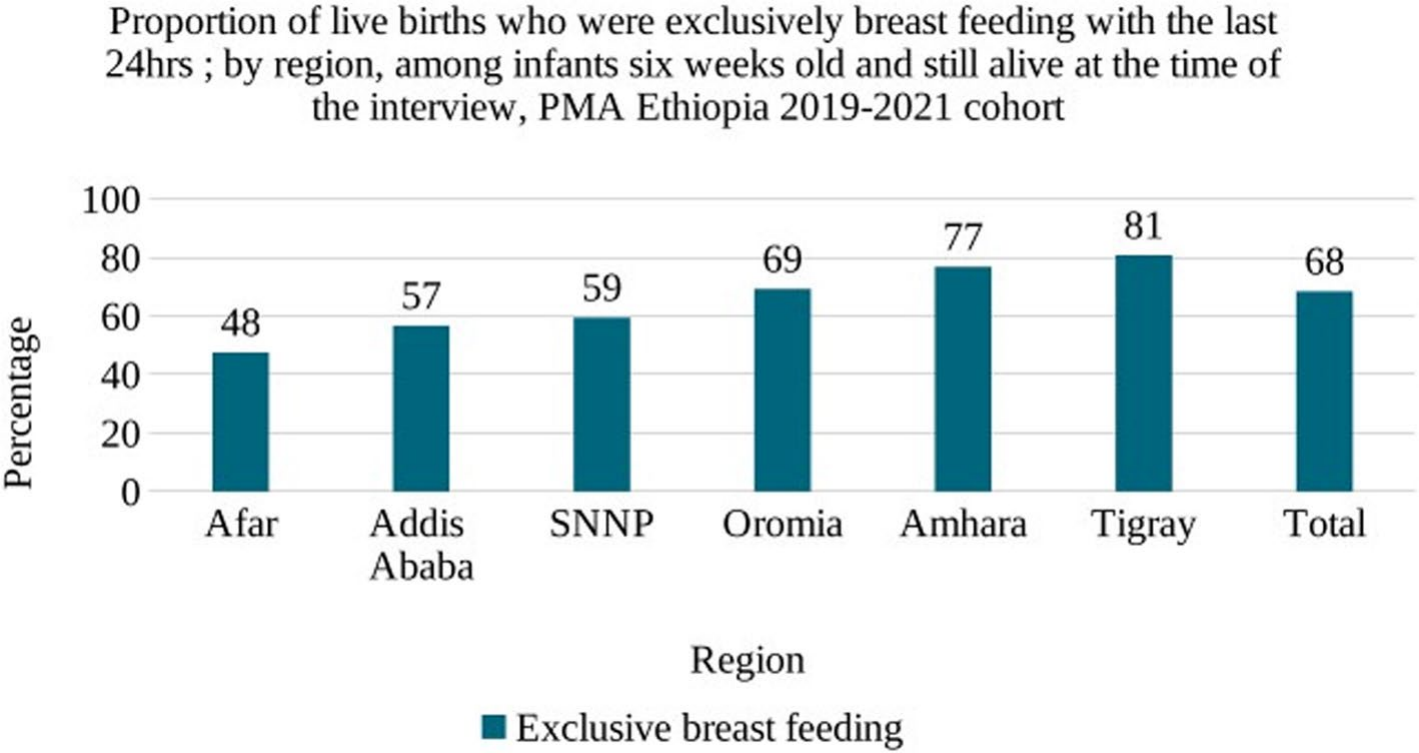
Proportion of live births who were exclusively breastfeeding with the last 24 h, by region: among infants six weeks old and still alive at the time of the interview, PMA Ethiopia 2019–2021 cohort

Nearly seven in ten (68.4%) women practiced EBF at six weeks postpartum with significant variation across regions, ranging from the lowest in Afar to the highest in Tigray region (Fig. 4).

### Determinants of early initiation of breastfeeding

The mode of delivery had significant association with timely initiation of breastfeeding with mothers delivering by caesarean section having 73% reduced odds of early breastfeeding initiation (AOR 0.27; 95% CI 0.17, 0.41) compared to mothers who had a vaginal delivery. In addition, mothers who have two or more children had a higher chance of initiating breastfeeding timely (AOR 1.54; 95% CI 1.13, 2.09) than primiparous women. The likelihood of early breastfeeding initiation were more than two times higher for mothers who were delivered by a skilled birth attendant (SBA) (AOR 2.13; 95% CI 1.01, 4.48) compared to mothers who did not have SBA assistance.

Mothers whose baby cried immediately after birth without a need of resuscitation were three times more likely to initiate breastfeeding timely (AOR 3.31; 95% CI 1.95, 5.62). Again, immediate skin-to-skin contact; putting the infant on the mother's chest right after delivery increased the likelihood to early initiation of breastfeeding (AOR 1.58; 95% CI 1.14, 2.20). In addition, timely breastfeeding initiation was also significantly associated with immediate PNC (PNC with in 1 h of delivery) where breastfeeding is more likely to start right after delivery for women who received immediate postnatal care than for mothers who didn't (AOR 1.54; 95% CI 1.07, 2.22).

Mothers who had obstetric complications during birth or within 24 h of birth have a lesser chance for early initiation of breastfeeding (AOR 0.74; 95% CI 0.58, 0.95). Women from the Afar region were less likely to adhere to the suggested breastfeeding initiation time compared to women from other regions (AOR 0.36; 95% CI: 0.14, 0.95).

Table 5 shows crude and adjusted odds ratios (AOR) that were calculated to determine the strength of association between the co-variates and early initiation of breastfeeding.

**Table 5:** Factors associated with early initiation of breastfeeding: results from unadjusted and adjusted logistic regression model, *among infants six weeks old and still alive at the time of the interview, PMA Ethiopia 2019–2021 cohort* (*n* = 2564)

	**COR (95% CI)**			***P*-Value**	**AOR (95% CI)**			***P*-Value**
**Maternal age**								
15–19 (ref)								
20 -24	1.34	(0.90	2.01)	0.152	1.07	(0.68	1.68)	0.776
25–34	1.07	(0.74	1.56)	0.707	0.83	(0.52	1.34)	0.446
35–49	0.90	(0.59	1.37)	0.611	0.69	(0.39	1.24)	0.216
**Maternal education**								
No education (ref)								
Primary	1.05	(0.82	1.35)	0.699	0.93	(0.69	1.24)	0.604
Secondary and higher	1.35	(0.99	1.83)	0.060	1.11	(0.75	1.66)	0.595
**Marital status**								
Married (ref)								
Unmarried	0.81	(0.52	1.26)	0.341	0.84	(0.52	1.35)	0.472
**Residence**								
Rural (ref)								
Urban	1.45	(1.03	2.04)	0.032	1.37	(0.84	2.25)	0.206
**Religion**								
Protestant (ref)								
Orthodox	0.89	(0.62	1.28)	0.541	0.73	(0.45	1.18)	0.197
Muslim	1.17	(0.77	1.79)	0.463	1.17	(0.69	1.98)	0.574
Catholic/ ~ s	0.67	(0.24	1.84)	0.435	0.50	(0.17	1.46)	0.207
**Parity**								
1 (ref)								
2 +	1.17	(0.94	1.45)	0.0.16	1.54	(1.13	2.09)	0.006*
**Wealth index**								
Low (ref)								
Middle	1.02	(0.74	1.40)	0.918	0.99	(0.70	1.40)	0.944
High	1.15	(0.85	1.56)	0.352	0.76	(0.51	1.12)	0.165
**ANC visit**								
0 visit (ref)								
1–3 visits	0.82	(0.62	1.09)	0.166	0.75	(0.55	1.03)	0.073
4 + visits	1.21	(0.91	1.59)	0.186	1.03	(0.74	1.41)	0.881
**Delivery place**								
Home (ref)								
Facility	1.76	(1.37	2.26)	0.000	0.73	(0.33	1.62)	0.432
**Mode of delivery**								
Vaginal (ref)								
Caesarean	0.27	(0.19	0.38)	0.000	0.27	(0.17	0.41)	0.000***
**Skilled birth attendant**								
No (ref)								
Yes	1.78	(1.38	2.28)	0.000	2.13	(1.01	4.48)	0.046*
**Baby cry/breathe normally immediately after birth**								
No (ref)								
Yes	9.78	(6.37	15.04)	0.000	3.31	(1.95	5.62)	0.000***
**Baby placed on mother’s chest immediately after delivery**								
No (ref)								
Yes	3.02	(2.39	3.82)	0.000	1.58	(1.14	2.20)	0.006**
**Obstetric complication**								
No (ref)								
Yes	0.70	(0.56	0.87)	0.001	0.74	(0.58	0.95)	0.017*
**Pregnancy intention**								
Unintended (ref)								
Intended	1.08	(0.86	1.36)	0.511	1.18	(0.91	1.52)	0.215
**Baby gender**								
Male (ref)								
Female	1.12	(0.91	1.39)	0.283	1.10	(0.88	1.37)	0.410
**PNC within 1 h of birth**								
No (ref)								
Yes	1.57	(1.15	2.15)	0.005	1.54	(1.07	2.22)	0.021*
**Maternity home**								
No (ref)								
Yes	1.15	(0.86	1.53)	0.345	1.11	(0.78	1.58)	0.577
**Region**								
Addis(ref)								
Tigray	0.91	(0.47	1.74)	0.767	0.96	(0.46	1.99)	0.911
Afar	0.45	(0.20	1.00)	0.049	0.36	(0.14	0.95)	0.040*
Amhara	0.69	(0.37	1.29)	0.248	0.81	(0.39	1.67)	0.565
Oromia	1.24	(0.67	2.29)	0.495	1.18	(0.58	2.40)	0.655
SNNP	1.09	(0.59	2.04)	0.781	1.16	(0.53	2.53)	0.709
**Random effect**								
**Var (EA)**	0.80					(0.54	1.18)	

^*^P < 0.05 ** *p* < 0.01 *** *p* < 0.001

### Determinants of exclusive breastfeeding (EBF)

The chance of exclusive breastfeeding were more than two times higher for mothers between the ages of 20 and 24 (AOR 1.48; 95% CI 1.01, 2.16) compared to the older age groups. In addition, mothers who had two or more children had a higher odd of exclusive breastfeeding compared to primiparous women (AOR 1.41; 95% CI 1.08, 1.82). Mothers whose baby cried immediately after birth without the need for resuscitation were more likely to exclusively breastfeed (AOR 1.81; 95% CI 1.09, 2.99). The baby’s sex had significant association, with female babies having an increased odds of exclusive breastfeeding (AOR 1.38; 95% CI 1.14, 1.67). Mothers whose child had some illness between delivery and six weeks postpartum have a reduced chance for early initiation of breastfeeding (AOR 0.57; 95% CI 0.47, 0.69). Women from Amhara (AOR 3.29; 95% CI 1.86, 5.84) and Tigray region (AOR 4.64; 95% CI 2.59, 8.31) was more likely to exclusively breastfed compared to women from other regions.

Table 6 shows crude and adjusted odds ratios (AOR) that were calculated to determine the strength of association between the co-variates and exclusive breastfeeding.

**Table 6:** Factors associated with exclusive breastfeeding/EBF: results from unadjusted and adjusted logistic regression model; among infants six weeks old and still alive at the time of the interview, PMA Ethiopia 2019–2021 cohort (*n* = 2564)

	**COR (95% CI)**			***P*-Value**	**AOR (95% CI)**		***P*-Value**
**Maternal age**							
15–19 (ref)							
Youth (20–24)	1.69	(1.19	2.39)	0.004	1.48	(1.01 2.16)	0.043*
Adult1(25–34)	1.54	(1.11	2.13)	0.009	1.18	(0.79 1.75)	0.419
Adult2(35–49)	1.40	(0.96	2.04)	0.084	0.99	(0.61 1.62)	0.969
**Maternal education**							
No education (ref)							
Primary	0.83	(0.67	1.04)	0.108	0.83	(0.64 1.07)	0.159
Secondary or higher	1.08	(0.83	1.42)	0.566	1.01	(0.72 1.41)	0.951
**Marital status**							
Married (ref)							
Unmarried	0.94	(0.63	1.43)	0.785	0.94	(0.61 1.45)	0.784
**Residence**							
Rural (ref)							
Urban	1.08	(0.80	1.46)	0.619	1.05	(0.703 1.574)	0.806
**Religion**							
Protestant (ref)							
Orthodox	1.46	(1.07	1.99)	0.016	0.76	(0.52 1.11)	0.154
Muslim	1.23	(0.86	1.76)	0.249	0.97	(0.64 1.46)	0.883
Catholic/Others	1.03	(0.43	2.45)	0.955	0.72	(0.29 1.74)	0.463
**Parity**							
1 (ref)							
2 +	1.27	(1.05	1.54)	0.014	1.41	(1.08 1.82)	0.01*
**Wealth index**							
Low (ref)							
Middle	1.29	(0.96	1.73)	0.091	1.29	(0.95 1.75)	0.107
High	1.24	(0.949	1.62)	0.115	1.13	(0.80 1.59)	0.493
**ANC visit**							
0 visit (ref)							
1–3 visits	1.27	(0.984	1.646)	0.066	1.16	(0.88 1.52)	0.303
4 + visits	1.40	(1.097	1.777)	0.007	1.22	(0.94 1.59)	0.138
**Delivery place**							
Home (ref)							
Delivery at facilities	1.10	(0.874	1.379)	0.422	0.98	(0.51 1.87)	0.940
**Mode of delivery**							
Vaginal (ref)							
Caesarean	0.82	(0.580	1.146)	0.239	0.97	(0.64 1.47)	0.897
**Skilled birth attendant**							
No (ref)							
Yes	1.07	(0.851	1.346)	0.562	1.05	(0.58 1.89)	0.885
**Baby cry/breathe normally immediately after birth**							
No (ref)							
Yes	5.25	(3.468	7.947)	0.000	1.81	(1.09 2.99)	0.020*
**Baby placed on mother’s chest immediately after delivery**							
No (ref)							
Yes	1.44	(1.179	1.761)	0.000	1.21	(0.92 1.60)	0.171
**Obstetric complication**							
No (ref)							
Yes	0.84	(0.686	1.015)	0.071	0.93	(0.75 1.14)	0.470
**Pregnancy intention**							
Unintended (ref)							
Intended	0.99	(0.805	1.211)	0.903	1.00	(0.80 1.25)	0.984
**Baby sex**							
Male (ref)							
Female	1.44	(1.191	1.733)	0.000	1.38	(1.14 1.67)	0.001**
**PNC**							
No (ref)							
Yes	0.94	(0.760	1.158)	0.552	0.80	(0.61 1.06)	0.123
**EBF counseling**							
No (ref)							
Yes	1.02	(0.793	1.308)	0.888	0.92	(0.71 1.21)	0.563
**Maternity home**							
No (ref)							
Yes	1.07	(0.829	1.383)	0.600	0.93	(0.69 1.24)	0.610
**Any illness**							
No (ref)							
Yes	0.65	(0.535	0.786)	0.000	0.57	(0.47 0.69)	0.000***
**Region**							
Addis (ref)							
Tigray	3.70	(2.188	6.267)	0.000	4.64	(2.59 8.31)	0.000***
Afar	0.68	(0.367	1.248)	0.211	0.58	(0.28 1.24)	0.159
Amhara	2.88	(1.748	4.738)	0.000	3.29	(1.86 5.84)	0.000***
Oromia	1.66	(1.036	2.644)	0.035	1.54	(0.89 2.63)	0.116
SNNP	1.09	(0.679	1.748)	0.721	1.08	(0.60 1.93)	0.808
**Random effect**							
**Var (EA)**	0.44					(0.27 0.69)	

^*^P < 0.05 ** *p* < 0.01 *** *p* < 0.001

## Discussion

This study aimed to assess early initiation of breastfeeding and exclusive breastfeeding and their determinants among infants of six-week age in Ethiopia. We found that more than three quarters of infants (77.8%) of six weeks old were introduced to breastfeeding within one hour of birth and nearly seven in ten (68.4%) of infants were exclusively breast fed at the six week postpartum period.

A similar rate of early initiation was reported by DHS in Ethiopia (73%) [14], Namibia (74.9%) [24] and Malawi (76.9%) [25] but higher compared to some other sub-Saharan and south east Asian countries. The variation is likely explained by the geographic, economic and cultural difference [11, 12]. Our finding for the rate of exclusive breastfeeding at the age of six week postpartum is close to EDHS finding, 64% at 2 to 3 month of age but higher than the 58% national figure [14]. This could be due to the fact that our data is collected shortly after birth at six-week postpartum period, and this likely reduces the risk of maternal recall bias, which may have impacted results from cross-sectional DHS surveys and other similar studies.

The result of this study revealed that mothers who had vaginal delivery were more likely to initiate breastfeeding within one hour of birth as compared to mothers who had caesarean section. This finding is comparable to previous studies [25, 26] and could be explained by the fact that cesarean delivery may introduce barriers, such as a delay in skin-to-skin contact between mother and child due to anesthesia or other medications.

Delivery assisted by a skilled birth attendant was also positively associated with early initiation of breastfeeding compared to those by traditional birth attendants (TBAs) or other non-health professionals’ assisted delivery. Similarly the practice of putting the baby skin-to-skin on mother’s chest immediately after birth significantly reduced the breastfeeding initiation time, which is consistent with findings from several other studies This finding is consistent with studies conducted in Ethiopia and south Asian countries [26–28].

The role of parity in lack of early initiation of breastfeeding may be related to some interplay between lack of knowledge and experience. In this study the odds of early breastfeeding initiation were almost two times higher for mothers who had two or more children compared to mothers who had one, which was consistent with findings from six low and middle-income countries [29] and Nigeria [10].

Compared to mothers who had no immediate postnatal care, mothers who got the PNC visit within 1 h of delivery initiated timely breastfeeding, possibly reflecting the impact of postpartum follow up services, including the promotion of the breastfeeding message by health professionals. This finding is similar to that of the study conducted in Bangladesh [12].

This study highlighted that complications during labor and within the first 24 h after delivery can negatively impact breastfeeding initiation time, possibly due to an increased postnatal pain from the complication, medications/analgesics or separation of mother and infant in the moments after birth. Furthermore, mothers whose babies needed resuscitation after birth were less likely to initiate breastfeeding immediately following delivery. This was supported by studies conducted in low and middle-income countries [29, 30].

This study revealed that maternal age and parity was significantly associated with exclusive breastfeeding practice. Mothers who were 20–24 years old were more likely to practice exclusive breastfeeding than those who were younger, which is a similar finding to that of a study conducted in West Oromia, Ethiopia [31]. In addition, the chance of exclusive breastfeeding was higher among moms who had two or more children. This might be due to the fact that mothers who has multiple children have more experience and knowledge on EBF when compared to primiparous women [29].

The sex of the infant was also one of the individual level factors significantly associated with exclusive breastfeeding The finding was consistent with studies done in Ethiopia, Cameroon and Angola [32]. The study showed that female infants were more likely to be exclusively breastfed compared to male infants. This may be related with the perception that breast milk alone does not meet their nutritional requirements and the belief that male infants have a more insatiable appetite and need additional food than female infants, which prompts the early introduction of complementary foods for male infants [33].

Infant comorbidity was negatively associated with exclusive breastfeeding which is concurrent with other studies [32, 34]. The possible justification might be that when the infant became sick, mothers may perceive that a change in the diet is essential to boost the energy and immunity of the infant. Furthermore, they may perceive additional foods may also be a treatment option for the sick infant.

Distribution of both early initiation of breastfeeding and exclusive breastfeeding rates vary across the regional states in Ethiopia. Similar to the EDHS finding [14], mothers from Afar region have a lesser chance for early initiation of breastfeeding. This could be related to the high prevalence of home delivery, as well as cultural and traditional beliefs, myths, and misconceptions about breastfeeding in this region [35]. Mothers from Tigray and Amhara had significantly higher odds of EBF than the others. This is supported by a EDHS analysis studies and could be explained by differences in culture, socioeconomic status and availability of health resources across the different regions of Ethiopia [36, 37].

One of the strengths of this study is that we used data from the PMA Ethiopia which is a national longitudinal survey collecting panel data from the six regions, thus enabling us to prospectively determine the rates of early initiation of breastfeeding and exclusive breastfeeding at six-week postpartum period, when mothers have a better recall.

Limitations of our study include, even though the time duration was not over months and years, we still rely on maternal recall and self-reporting about precisely when they initiated breastfeeding and whether they exclusively breastfed in the day prior to the survey. Furthermore, as the PMA panel survey did not cover all the regions, the regional-level analysis can only be inferred to the six regions included in the survey.

## Conclusions

The rate of early initiation of breastfeeding and exclusive breastfeeding in Ethiopia are improving in comparison to the global benchmark, but they remain suboptimal and below the national target. Early initiation of breastfeeding and EBF in Ethiopia are associated with various sociodemographic, maternal and child health service characteristics. Intervention strategies should target the regional variations, nulliparous women, mothers with caesarean deliveries, obstetric complications and neonatal illness/care. In addition, further research (qualitative study) is required to better understand the causes for the regional variations in these breastfeeding patterns. Those factors associated with these key breastfeeding indicators should assist with more effective strategies to scale-up breastfeeding interventions.

## Data Availability

The datasets generated during the study are publicly available from the PMA website. https://www.pmadata.org/data/request-access-datasets.

## Abbreviations

AIC: Akaike Information Criteria
ANC: Antenatal care
AOR: Adjusted odds ratio
CI: Confidence interval
COR: Crude odds ratio
DHS: Demographic and Health Survey
EBF: Exclusive Breastfeeding
EDHS: Ethiopian Demographic and Health Survey
LBW: Low birthweight
PMA: Ethiopia: Performance Monitoring for Action Ethiopia
PNC: Postnatal care
SNNP: Southern Nations, Nationalities, and Peoples
WHO: World Health Organization
